# RNAi Screening in Tumor Cells Identifies Artificial microRNAs That Improve Oncolytic Virus Replication

**DOI:** 10.3390/ph18050708

**Published:** 2025-05-10

**Authors:** Hannah Klemets, Angelina Bardoul, Adrian Pelin, Ragunath Singaravelu, Meaghan Boileau, Theresa Falls, Julia Petryk, Marie-Claude Bourgeois-Daigneault, John C. Bell, Dominic G. Roy

**Affiliations:** 1 Cancer Axis, Centre de Recherche du Centre Hospitalier de l’Université de Montréal, Institut du Cancer de Montréal, Montreal, QC H2X 0A9, Canada; 2 Department of Microbiology, Infectious Diseases and Immunology, Faculty of Medicine, University of Montreal, Montreal, QC H3T 1J4, Canada; 3 Centre for Innovative Cancer Therapeutics, Ottawa Hospital Research Institute, Ottawa, ON K1H 8L6, Canada; 4 Department of Biochemistry, Microbiology and Immunology, University of Ottawa, Ottawa, ON K1H 8M5, Canada

**Keywords:** oncolytic virus, VSV, RNA interference, cancer

## Abstract

**Background/Objectives**: Oncolytic viruses infect and kill tumor cells while leaving normal cells unharmed. They are often attenuated through the reduction in their ability to antagonize antiviral defenses, leading to robust replication in tumor cells, which often possess defects in antiviral pathways, while minimizing replication in normal cells. However, not all tumors have defects in their antiviral defenses, and virus replication in these tumors is minimal, thus limiting therapeutic benefits. Therefore, identifying and modulating host factors that regulate virus replication in oncolytic virus-resistant cancer cells, but not normal cells, could lead to increased replication in these tumors and potentially improved therapeutic outcomes. **Methods**: To identify host factors that modulate oncolytic virus replication in tumor cells, we conducted an RNA interference screen by using a replication-competent library of Sindbis virus recombinants individually enabled with the capacity to elicit RNA interference in host genes via the expression of artificial microRNAs. Since the expression of artificial microRNAs is coupled to virus replication, this results in the selective enrichment of viral clones which express an artificial microRNA that promotes virus replication. **Results**: By using this approach, the serial passage of the Sindbis virus–artificial microRNA library in a tumor cell line followed by the deep sequencing of the selected viral populations led to the identification of several artificial microRNA sequences that were enriched. Furthermore, the identified artificial miRNA sequences increased the replication of several oncolytic viruses both in vitro and in vivo, ultimately leading to an enhanced therapeutic effect. **Conclusions**: Altogether, our study highlights the utility of this screening platform in identifying artificial microRNAs that enhance oncolytic virus efficacy.

## 1. Introduction

Oncolytic viruses (OVs) are multi-modal biological therapeutics with the ability to directly infect and kill tumor cells while also stimulating potent anti-tumor immune responses [1,2]. With the capacity to engineer OVs to express therapeutic transgenes that improve virus replication [3,4], increase tumor killing [5,6], or enhance their immuno-stimulatory functions [7,8,9], OVs can deliver a multi-pronged attack against cancer. The clinical success of an oncolytic herpes simplex virus (T-VEC) has fueled excitement in the field [10,11,12,13], and several OVs are currently undergoing pre-clinical and clinical testing as standalone therapies or in combination with other therapeutic modalities [14]. Vesicular stomatitis virus (VSV) is a clinical trial candidate (NCT01628640 and NCT02923466) that is being investigated for a variety of cancer types. A variant of the virus, VSVΔ51, has been engineered to lack the ability to block the host antiviral interferon (IFN) response [15,16], making it highly attenuated in normal cells, which have an intact IFN pathway, and yet highly lytic in cancer cells, which often have defects in this pathway [17]. This is achieved by the deletion of methionine 51 (VSV-Δ51) in its matrix protein, which is responsible for the inhibition of the IFN response [15,16].

While impressive patient responses have been reported, thus supporting the therapeutic benefit of OVs, there are many aspects of the therapy that remain poorly understood, and not all patients respond to the treatment. One of the factors that can limit OV treatment efficacy is the inherent susceptibility of tumors to infection, with some tumors supporting robust virus replication, and others being completely refractory. Indeed, the ability of viruses to successfully replicate within their host depends on several cellular pathways, many of which can in turn modulate virus replication. Therefore, pre-clinical studies aimed at understanding the cellular factors that influence the effectiveness of OV therapy could lead to the development of improved viruses and novel therapeutic strategies aimed at increasing their replication and cytotoxicity in tumor cells.

The discovery of RNA interference (RNAi) has led to the development of several strategies and technologies that harness its ability to modulate gene expression to treat various diseases, including cancer. For example, siRNAs and shRNAs can be used to modulate the expression of a given target gene, while microRNA sponges can be used to negate the unwanted effects of specific microRNAs [18]. In the context of antiviral immunity, one approach to identifying cellular factors that regulate virus replication on a genome-wide scale is RNAi-based screening. RNAi screens have uncovered the functions of several antiviral genes [19,20,21] that regulate various aspects of virus infection, including entry, uncoating, replication, and budding, for a wide range of viruses, such as VSV, human immunodeficiency virus, dengue, hepatitis C virus, and influenza virus [22,23,24,25,26]. This approach has also been utilized to successfully uncover cellular factors that regulate OV efficacy in tumor cells [27]. Furthermore, OVs can be engineered to express small RNAs (miRNAs and shRNAs) to achieve the desired therapeutic effect, such as increasing virus replication, cytotoxicity, or the remodeling of the tumor microenvironment [28,29,30,31,32,33,34]. For example, herpes simplex virus (HSV) has been engineered to express an shRNA targeting the antiviral factor ISG15 to promote virus replication [35], and adenovirus has been engineered to express an shRNA against PD-L1 to increase anti-tumor immune responses [36]. A genome-wide screening approach can be applied by using a pool of viruses each encoding an artificial miRNA (amiRNA) and thus enabled with the capacity to modulate gene expression [19,28,37]. Since the expression of the artificial microRNA is coupled to virus replication, this results in the selective enrichment of viral clones which express an artificial microRNA that promotes virus replication.

The tumor selectivity of OVs has been attributed to various molecular features unique to tumor cell biology. For IFN-sensitive viruses such as VSV and Sindbis virus (SINV), tumor-specific defects in the IFN pathway underlie tumor-selective replication [15,17,38]. Additional features common to most cancers, such as activated growth pathways, elevated nucleotide pools, and increased protein synthesis, can also promote OV replication [39]. Given that tumor cells committed to the malignant phenotype have already differentiated themselves from their normal counterparts with respect to their ability to resist virus replication, they may be uniquely sensitized to OVs by the knockdown of particular antiviral gene products. Therefore, screening the SINV–amiRNA library on tumor cells could reveal antiviral gene products that are redundant in normal tissues but indispensable in malignant cells.

In the current study, we demonstrate that the passage of a SINV–amiRNA library in tumor cells resulted in the enrichment of specific amiRNA-expressing SINV clones. Furthermore, the identified amiRNA sequences increased the replication of several OVs both in vitro and in vivo, ultimately leading to improved tumor control. Taken together, these data suggest that this screening approach can be implemented to generate OVs with enhanced therapeutic activity.

## 2. Results

### 2.1. In Vitro Serial Passage of SINV–amiRNA Library in Tumor Cells Selects for Specific amiRNAs

**Figure 1:**
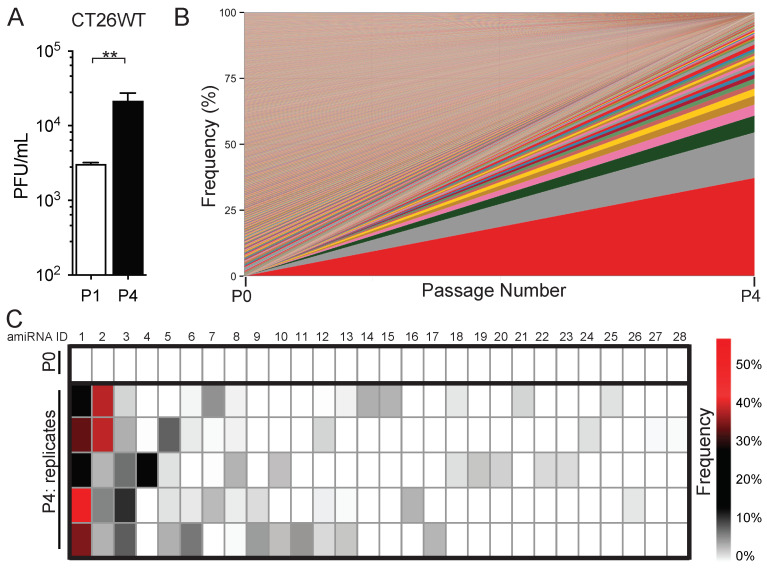
Enrichment of specific amiRNA sequences after serial passage of SINV library in tumor cells. (**A**) SINV titers obtained after 1 passage (P1) and 4 serial passages (P4) in CT26WT tumor cells infected at multiplicity of infection (MOI) of 0.1 for 48 h (*n* = 5). Data are presented as means ± standard error of the mean (SEM). Student’s unpaired, one-tailed *t*-test was performed; **: *p* < 0.01. (**B**) Stacked area plot of percent frequency of each amiRNA in SINV–amiRNA library before passaging (P0) and after 4 rounds of serial passage (P4) in CT26WT cells. Cumulative percent frequencies of all 5 replicates are shown for P4. Each color represents an individual amiRNA. (**C**) Heat map showing percent frequency of reads of the 10 most enriched amiRNAs in each replicate at P0 (input) and after P4.

The mammalian antiviral response involves the coordination of extensive signaling networks that result in the production of hundreds of antiviral genes. We, therefore, performed a genome-wide RNAi-based screen in which amiRNA expression was coupled to virus replication with the notion that viruses expressing an amiRNA that targets host factors regulating virus replication will have a growth advantage. Therefore, the serial passage of a library of viruses each expressing a unique amiRNA should result in the enrichment of specific amiRNAs that increase virus replication. To this end, we passaged the SINV–amiRNA library [19] in CT26WT murine colon carcinoma cells for a total of four rounds and sequenced the resulting viral populations to assess amiRNA enrichment. We observed that virus output from the tumor cells increased from passage 1 to passage 4, suggesting that the enriched SINV clones have a growth advantage over the initial pool that was used for the first passage (Figure 1A). The deep sequencing of passage 4 revealed that several amiRNA sequences were indeed enriched compared with the original, un-passaged library (Figure 1B, Table S1).

Importantly, a total of 56 amiRNAs were present at frequencies above 1%, with 15 of these amiRNAs being found in two replicates, 2 of them present in four replicates, and another 2 being found in all five replicates, indicating that the enrichment of these particular amiRNA-expressing virus clones was likely mediated by the amiRNA and not due to unrelated mutations in the virus backbone that were positively selected for (Figure 1C). To confirm the sequencing results, we picked 20 viral plaques at random from P4 and performed a PCR screen for the two most enriched amiRNA sequences and found that of the 20 plaques picked, 4 plaques (20%) corresponded to amiRNA-1 and 1 plaque (5%) corresponded to amiRNA-2, which is consistent with the RNA sequencing data.

### 2.2. Enriched amiRNA Sequences Increase SINV Replication in Tumor Cells

**Figure 2:**
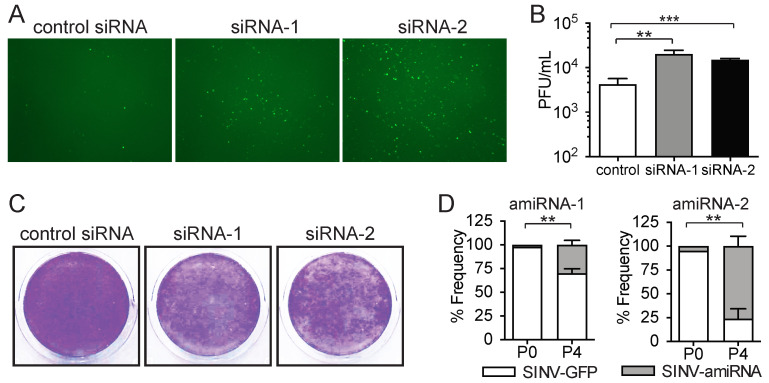
Enriched amiRNAs increase SINV replication. (**A**) Fluorescent images (4× magnification) and (**B**) virus titers from CT26WT cells transfected with siRNAs corresponding to amiRNA-1 and -2 targeting sequences and subsequently infected with SINV-GFP at MOI of 0.1. Fluorescent images were taken, and supernatants were collected 48 h post-infection (*n* = 4). (**C**) Virus-induced cytotoxicity revealed by Coomassie Blue stain of CT26WT cells transfected with siRNAs corresponding to identified hits and subsequently infected with SINV-GFP. Cells were fixed and stained 72 h post-infection. (**D**) Enrichment of amiRNA-expressing SINV from competition experiments against parental SINV-GFP virus. The percentage of each virus is shown before competition (P0) and after 4 rounds of competition (P4) in CT26WT cells (*n* = 3). Data are presented as means ± SEM. Student’s unpaired, one-tailed *t*-test was performed; **: *p* < 0.01; ***: *p* < 0.001.

We chose to focus on the two most enriched sequences, as these were highly enriched relative to the other sequences in all biological replicates (Figure 1C and Table S1). To confirm that the amiRNA sequences we identified as enriched after serial passaging in tumor cells did in fact increase SINV replication, we transfected CT26WT cells with siRNAs corresponding to the target sequences of the identified amiRNAs and subsequently infected them with SINV-GFP. Increased virus replication and spread within the tumor cells were evident, as assessed by the fluorescence imaging of green fluorescent protein (GFP) reporter expression by the virus (Figure 2A). The quantification of virus output by the plaque assay revealed that replication was increased approximately 5-fold in cells transfected with siRNA-1 and -2 compared with a control siRNA targeting luciferase, which is not expressed by our cells (Figure 2B). This increase in SINV replication also resulted in greater tumor cell killing (Figure 2C).

Despite the finding that the siRNA transfection of the identified sequences can increase virus replication and tumor cell killing, this experimental approach results in the knockdown of the target genes before virus infection, whereas gene knockdown occurs after virus replication is initiated with the SINV–amiRNA library screening approach. We, therefore, sought to confirm that as suggested by our screen, SINV-mediated amiRNA expression could also increase virus replication. We, therefore, cloned the identified amiRNAs back into the parental SINV virus to exclude that any mutations in the virus backbone that may have appeared during the passage could have confounded our analysis and examined if the amiRNA-expressing viruses replicated better than the parental GFP-expressing virus in a competition assay. To do so, SINV–amiRNA viruses and the parental SINV-GFP were mixed in a ratio of 1:100 and used to infect CT26WT cells, and the frequency of GFP-positive plaques in 100 randomly selected plaques was quantified. Our data show that the amiRNA viruses were initially present at frequencies of only 2% and 5% (Figure 2D). Interestingly, after four serial passages in CT26WT cells, the frequencies of SINV–amiRNA-1 and SINV–amiRNA-2 had increased to 30% and 76%, respectively, suggesting that they do indeed confer a growth advantage to the virus (Figure 2D). We performed a similar experiment in human HCT116 colon cancer cells, and consistent with the competition experiment in the CT26WT murine cells, amiRNA-expressing viruses also increased in frequency after four rounds of competition (Figure S1). Importantly, amiRNA expression was maintained throughout multiple rounds of infection, suggesting that the amiRNA-expressing viruses are stable and do not lose expression of the transgene. Overall, these results confirm that the enriched amiRNAs identified in our screening approach increase SINV replication.

### 2.3. amiRNA-1 and amiRNA-2 Enhance Replication and Tumor Cell Killing of Other OVs

**Figure 3:**
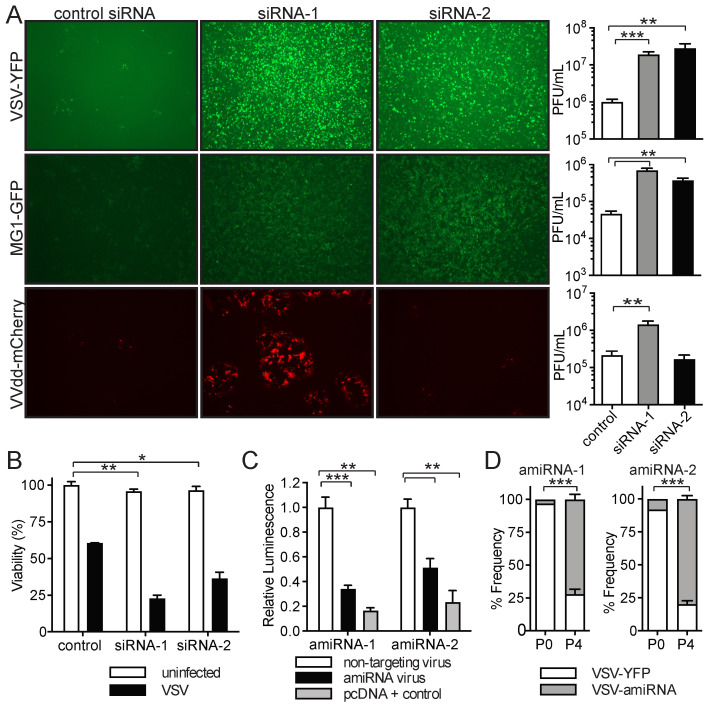
The identified amiRNAs increase the replication of oncolytic VSVΔ51, MG1, and VVdd. (**A**) Fluorescent images (4× magnification) and virus titers from CT26WT cells transfected with siRNAs corresponding to the identified hits and subsequently infected with VSVΔ51-YFP (top panel, *n* = 6), MG1-GFP (middle panel, *n* = 3), or VVdd-mCherry (bottom panel, *n* = 5). Images were taken and supernatants were collected 48 h post-infection for VSVΔ51 and MG1 and at 72h for VVdd. (**B**) Virus-induced cytotoxicity as measured by AlamarBlue viability assay of CT26WT cells transfected with siRNAs corresponding to identified hits and subsequently infected with VSVΔ51-YFP. Cell viability was measured 72 h post-infection (*n* = 3). (**C**) Dual-luciferase reporter assay. BHK cells were transfected with the indicated reporter plasmids possessing target sites for enriched amiRNAs in 3′ UTR of Renilla luciferase reporter and subsequently infected with the indicated VSV viruses. Luciferase activity was measured 24 h post-infection. (*n* = 3). (**D**) Enrichment of amiRNA-expressing VSVΔ51 from competition experiments against parental virus VSVΔ51-YFP. The frequency of each virus is shown at P0 and P4 in CT26WT cells (*n* = 3). Data are presented as means ± SEM. Student’s unpaired, one-tailed *t*-test was performed; *: *p* < 0.05; **: *p* < 0.01; ***: *p* < 0.001.

Although it has been demonstrated that SINV has oncolytic properties [38,40,41,42,43], we wanted to determine if the amiRNAs we identified in our SINV library screen could also be exploited to increase the replication of other OVs. We, therefore, performed similar experiments with the OVs VSVΔ51-YFP, Maraba-MG1-GFP [44], and Vaccinia virus (VVdd [45]) expressing mCherry. The transfection of siRNAs corresponding to the identified amiRNA sequences followed by infection led to increases in virus replication in most cases, as assessed by florescent reporter gene expression and the qPCR analysis of viral genomes (Figure 3A and Figure S2). In line with the enhanced oncolytic activity, the quantification of virus outputs revealed that VSVΔ51 replication was enhanced more than 15-fold for both siRNA-1 and siRNA-2 (Figure 3A, top panels), while siRNA-1 and siRNA-2 increased MG1 replication 15-fold and 8-fold, respectively (Figure 3A, middle panels). Interestingly, VVdd replication was enhanced approximately 7-fold by siRNA-1, whereas siRNA-2 had no effect on virus replication (Figure 3A, bottom panels). Notably, the increase in VSVΔ51 replication also led to greater tumor cell killing (Figure 3B).

To confirm the positive impact that virus-mediated amiRNA expression has on virus replication, we cloned the enriched amiRNAs into VSVΔ51. We first confirmed that the VSVΔ51-mediated expression of amiRNAs resulted in the production of functional amiRNAs capable of silencing gene expression. By using a luciferase reporter assay where amiRNA target sites were introduced in the 3′ UTR of the luciferase gene, we found that the amiRNAs expressed by VSVΔ51 were able to effectively silence luciferase expression, whereas control virus expressing an unmatched amiRNA could not (Figure 3C and Figure S3). We then performed a virus competition assay to determine if VSVΔ51-amiRNAs had a growth advantage over the parental YFP-expressing virus. Before the serial passage, VSVΔ51-amiRNA-1 and VSVΔ51-amiRNA-2 viruses were present at frequencies of 3% and 8%, respectively. After four serial passages in CT26WT cells, the frequencies of amiRNA-expressing viruses had increased to 72% and 79%, indicating a clear growth advantage relative to the parental virus (Figure 3D). Therefore, the amiRNAs identified by the SINV–amiRNA library screening approach are capable of increasing the replication of other clinically relevant OVs.

### 2.4. amiRNA-1 and amiRNA-2 Enhance VSV Replication in an IFN-Independent Manner

**Figure 4:**
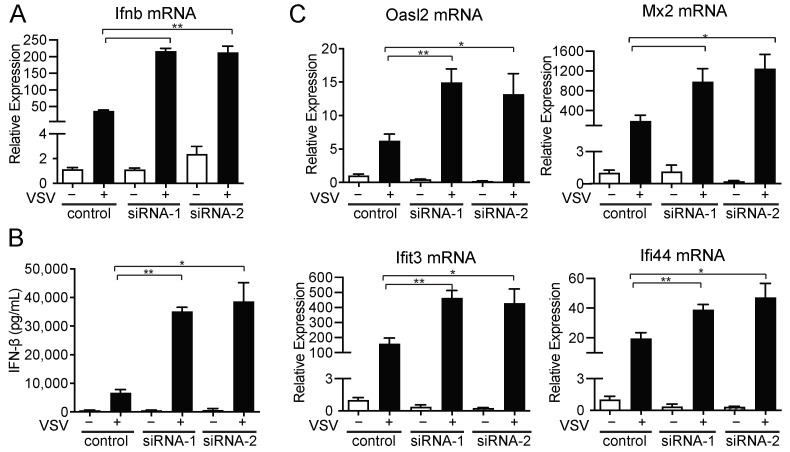
siRNA-1 and -2 do not target the IFN pathway. (**A**) IFN-β levels in CT26WT cells transfected with the indicated siRNA and then infected with VSVΔ51 at an MOI of 0.001 for 24h, as measured by qPCR (*n* = 2) and (**B**) ELISA (*n* = 2). Data are presented as means ± SEM. Student’s unpaired, one-tailed *t*-test was performed; *: *p* < 0.05; **: *p* < 0.01. (**C**) Relative expression of various ISGs after siRNA transfection and infection of CT26WT cells with VSVΔ51 at an MOI of 0.001 for 24h, as measured by qPCR (*n* = 3). Data are presented as means ± SEM. Student’s unpaired, one-tailed *t*-test was performed; *: *p* < 0.05; **: *p* < 0.01.

To understand how the identified amiRNAs were enhancing virus replication, we first looked at the ability of the cells to produce IFN-β, a key regulator of antiviral immunity against VSV and SINV, as well as several other viruses, following VSVΔ51 infection. As expected, CT26WT cells transfected with control siRNAs and subsequently infected with VSVΔ51 were able to induce IFN-β expression at both the mRNA and protein levels, as determined by RT-qPCR and ELISA performed on culture supernatants, respectively (Figure 4A,B). Similarly, when CT26WT tumor cells were transfected with siRNA-1 or siRNA-2 and infected with VSVΔ51, they too were able to produce IFN-β, albeit at higher levels than the control (Figure 4A,B). The enhanced IFN-β production is likely due to the fact that these cells were infected with VSVΔ51 at higher levels (Figure 3A), as we often observed a positive correlation between VSVΔ51 infection and IFN production (Figure S4). We next assessed whether the cells had functional type I IFN signaling by looking at their ability to induce the expression of several known interferon-stimulated genes (ISGs). CT26WT cells transfected with control siRNA and infected with VSVΔ51 were able to upregulate the expression of all ISGs tested (Figure 4C). Importantly, CT26WT cells transfected with siRNA-1 or siRNA-2 were able to induce all ISGs tested at even higher levels than the control siRNA, consistent with the increased levels of IFN-β produced by these cells and indicating proper IFN signaling (Figure 4A,B). Thus, our data show that amiRNA-1 and -2 do not affect the ability of cells to produce or respond to IFN-β.

**Figure 5:**
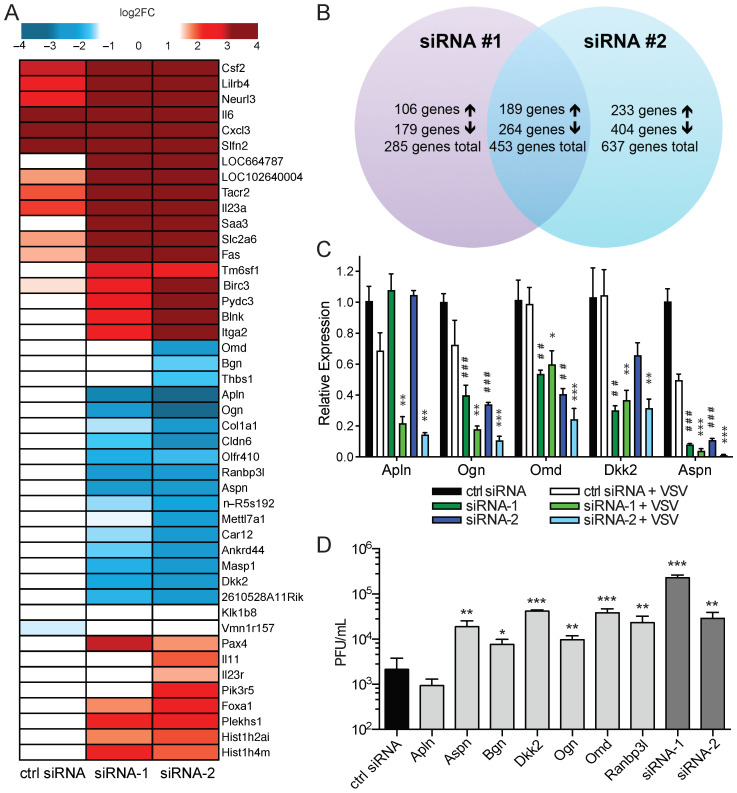
Gene expression changes induced by siRNA-1 and siRNA-2 reveals marked overlap of differentially expressed genes. (**A**) Microarray analysis of changes in gene expression 16h after VSVΔ51 infection of CT26WT transfected with siRNA-1 or siRNA-2. Heat map shows genes that were differentially expressed at least 3.5-fold compared with cells treated with control siRNA and infected with VSVΔ51. (**B**) Venn diagram showing number and overlap of differentially expressed genes (≥2-fold) in VSVΔ51-infected, siRNA-1- or siRNA-2-transfected cells, compared with control siRNA. Up arrows and down arrows indicate an increase and a decrease in expression, respectively. (**C**) qPCR analysis of selected genes identified as downregulated by microarray analysis of siRNA-treated CT26WT cells (*n* = 3). Data are presented as means ± SEM. One-way ANOVA was performed with Dunnett’s multiple comparisons post hoc test. Compared with control siRNA, ##: *p* < 0.01; ###: *p* < 0.001. Compared with control siRNA + VSV, *: *p* < 0.05; **: *p* < 0.01; ***: *p* < 0.001. (**D**) Knockdown of target gene promotes VSV replication in CT26WT cells. CT26WT cells were transfected with the indicated siRNAs, and 48 h post-transfection, they were infected with VSVΔ51. Supernatants were collected 48 h post-infection, and virus was quantified (*n* = 3). Data are presented as means ± SEM. Student’s unpaired, one-tailed *t*-test was performed; compared with control siRNA, *: *p* < 0.05; **: *p* < 0.01; ***: *p* < 0.001.

In an effort to identify which genes or pathways are affected by the enriched amiRNAs, we performed a microarray analysis of CT26WT cells transfected with control siRNA, siRNA-1, or siRNA-2 with and without VSVΔ51 infection (Figure 5A). Remarkably, there was significant overlap (*p* < 0.0001, Fisher’s exact test) in the genes differentially expressed (>2-fold) in siRNA-1- and siRNA-2-treated cells (Figure 5A,B), suggesting that amiRNA-1 and -2 may target similar genes or pathways.

Of the 738 (siRNA-1) and 1090 (siRNA-2) differentially expressed genes in VSV-infected, siRNA-1- and siRNA-2-transfected cells, respectively, 456 were common to both siRNAs. A qPCR analysis of select differentially expressed genes confirmed the differences observed in the microarray (Figure 5C). We focused our attention on the genes that were the most downregulated in both siRNA-1- and siRNA-2-treated cells, namely, Apln, Aspn, Bgn, Dkk2, Ogn, Omd, and Ranbp3l. The individual siRNA knockdown of these genes revealed that siRNAs targeting Aspn, Bgn, Dkk2, Ogn, Omd, and Ranbp3l increased VSV replication 3.5-fold to 18-fold, while the siRNA knockdown of Apln had no impact on VSV replication (Figure 5D). We also included siRNA-1 and siRNA-2 for comparison, and these increase VSV replication to an equal or greater extent than the knockdown of the individual genes (Figure 5D). These results suggest that several of the genes that are modulated by siRNA-1 and siRNA-2 are involved in regulating VSV replication in CT26WT cells.

### 2.5. amiRNA-Expressing Viruses Demonstrate Enhanced Replication and Efficacy In Vivo

**Figure 6:**
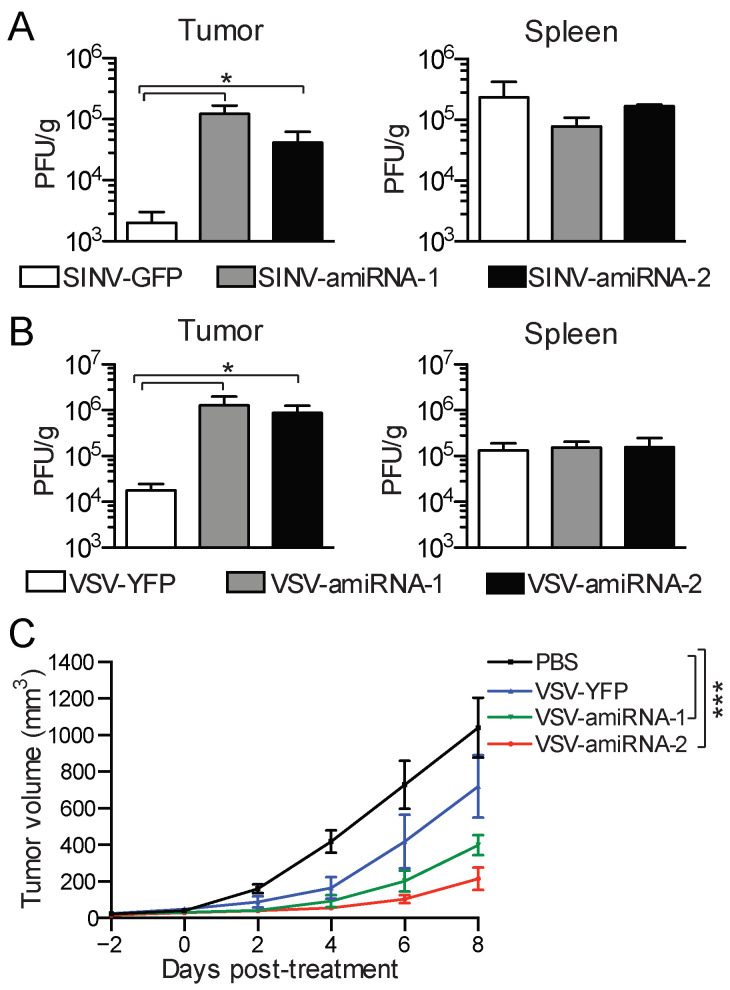
The amiRNA-expressing viruses show enhanced replication and efficacy in vivo. (**A**) Quantification of SINV in tumors (*n* = 6) and spleens (*n* = 3) 48 h post-intratumoral treatment of CT26WT tumor-bearing mice with 1 × 10^8^ PFU. Data are presented as means ± SEM. Student’s unpaired, one-tailed *t*-test was performed; *: *p* < 0.05. (**B**) Quantification of VSVΔ51 in tumors (*n* = 14) and spleens (*n* = 3) 48 h post-intravenous treatment of CT26WT tumor-bearing mice with 5 × 10^8^ PFU. Data are presented as means ± SEM. Student’s unpaired, one-tailed *t*-test was performed; *: *p* < 0.05. (**C**) Tumor volumes of mice (*n* = 6) bearing CT26WT subcutaneous tumors treated intravenously on day 1 and intratumorally on days 2 and 3 with 5 × 10^8^ PFU of the indicated viruses. Values represent means ± SEM. Two-way ANOVA test was performed with Dunnett’s multiple comparisons post hoc test; ***: *p* < 0.001.

We next determined if the in vivo replication of amiRNA-expressing viruses was also enhanced in CT26WT tumor-bearing mice. We initially looked at SINV–amiRNA replication in subcutaneous tumors after intratumoral injection, as well as in the spleen, to assess if virus-mediated amiRNA expression also impacted virus replication in normal tissues. In line with what we observed in our in vitro studies, the amiRNA-expressing viruses replicated to titers at least 30 times greater than that of the control virus within 48 h after injection (Figure 6A, left panel). Importantly, we did not find any differences in virus titers in the spleens of the mice, suggesting that the safety profiles of the viruses were not impaired (Figure 6A, right panel). We next performed similar experiments with VSVΔ51 amiRNA-expressing viruses. We measured virus titers in the tumors 48 h after injection and found that the amiRNA-expressing viruses replicated to titers 50 times higher than control virus (Figure 6B, left panel). Once again, we assessed virus titers in the spleen and observed no significant differences between the control virus and the amiRNA-expressing viruses (Figure 6B, left panel).

Finally, we determined whether the increase in the replication of the amiRNA-expressing VSV viruses we observed both in vitro and in vivo translates to enhanced therapeutic activity. To test this, we established subcutaneous CT26WT tumors in mice and treated them with the different viruses and monitored tumor growth. Although VSVΔ51-YFP was able to control tumor growth compared with no treatment, the delay in tumor progression was even more pronounced with VSVΔ51-amiRNA-1 and VSV-amiRNA-2 (Figure 6C). These data demonstrate that the enhanced replication conferred by amiRNA expression translates into better tumor control.

## 3. Discussion

Here, we show that the screening of a replication-competent SINV–amiRNA library results in the selection of amiRNA sequences that increase virus replication specifically in tumor cells. After as few as four passages in tumor cells, amiRNAs that were initially present at frequencies below 0.04% were enriched to frequencies as high as 58%. This enrichment is a combination of positive selection for amiRNAs that enhance SINV replication, as well as negative selection against amiRNAs that are detrimental to virus replication. It is also important to note that the kinetics of amiRNA expression are governed by SINV’s replication cycle. This certainly impacts the type of amiRNAs that are enriched during the screen, as amiRNAs that target genes encoding proteins with a half-life longer than the virus life cycle may not be enriched. Similarly, amiRNAs that target genes involved in regulating virus entry or uncoating would not be selected for, as these events precede viral gene transcription, amiRNA expression, and gene knockdown. This is not the case with classic siRNA- or shRNA-based screens, where the knockdown of genes occurs before virus infection is initiated; thus, genes regulating virus entry can be uncovered [46].

We chose to focus on the two most enriched amiRNAs, as these were found in all five biological replicates. Some of the amiRNAs were enriched but in only one or two of the replicates. This may be due to mutations that occur in the virus backbone that were positively selected for and therefore unrelated to amiRNA expression. For this same reason, it was important to re-clone the identified amiRNAs into the parental virus to effectively reset the viral genome when evaluating the impact of the amiRNAs on virus replication.

In our in vitro experiments, siRNA knockdown resulted in larger increases in virus replication compared with virus-mediated amiRNA expression. This is most likely because siRNAs delivered prior to infection allow for a more efficient knockdown of the relevant target genes. However, a recent study suggests that the pre-conditioning of uninfected cells by amiRNAs may also be occurring with virus-mediated amiRNA expression [28]. It was found that virus-infected cells released small extracellular vesicles containing virus-derived amiRNAs, which effectively primed neighboring cells for infection via a bystander effect. Interestingly, with the identification of sequence motifs that favor miRNA incorporation into extracellular vesicles [47] and the potential of expressing multiple amiRNAs at the same time, it may be possible to develop strategies using amiRNA-enabled OVs to reprogram the tumor microenvironment to achieve certain therapeutic outcomes.

In vivo, virus-mediated amiRNA expression resulted in larger increases in virus replication than in vitro, most likely due to the fact that selective pressure against the virus is greater in vivo and thus increases in viral fitness are more pronounced. Nonetheless, the potential to pre-condition the tumor for viral infection with the in vivo delivery of tumor-targeted siRNAs [48,49,50] is an approach worth investigating that may result in even more dramatic increases in virus replication compared with virus-mediated amiRNA expression. This is particularly relevant for VV, since the virus-mediated expression of amiRNAs is not a viable strategy to increase replication since the virus encodes a protein, VP55, which is known to polyadenylate various RNA species, including microRNAs, resulting in their degradation [51].

Although the screen was performed with SINV, a positive-sense RNA virus, the identified amiRNAs were also able to increase the replication of oncolytic VSVΔ51 and Maraba-MG1, which are negative-sense RNA viruses. These findings suggest that amiRNAs target antiviral factors that antagonize both viruses. This is not surprising given that both viruses are IFN-sensitive and that several ISGs can act on a wide range of viruses [20,52,53]. Interestingly, the replication of VV, a double-stranded DNA virus that encodes a vast array of immuno-modulatory proteins, was also increased but only with siRNA-1, suggesting that a subset of target genes of amiRNA-1 are different than those of amiRNA-2. Importantly, the amiRNA-expressing SINVs also replicated better in HCT116 cells, indicating that the amiRNAs can increase SINV replication in human tumor cells and suggesting that the relevant mRNA targets may also be expressed in human tumor cells and regulate virus replication. However, additional experiments would need to be performed to confirm this.

The mechanisms by which amiRNAs increase virus replication remain to be determined. We focused our initial analysis on the IFN pathway and observed more IFN-β mRNA and protein production, as well as higher ISG expression following infection with siRNA knockdown in tumor cells. These results suggest that the IFN pathway is not targeted by the amiRNAs but does not rule out the possibility that other pertinent ISGs may be affected. Surprisingly, gene expression analysis revealed a marked overlap in the genes affected by both siRNAs, suggesting that these siRNAs may function similarly to increase virus replication, at least in the case of VSV. Notably, 4 of the 17 members of the small leucine-rich proteoglycan (SLRP) gene family [54,55] (*Omd*, *Ogn*, *Aspn*, and *Bgn*) were found to be downregulated by both siRNAs, suggesting that these genes all have target sites in their mRNAs, or that they are regulated by a common transcription factor that is also a target of the siRNAs. The siRNA knockdown of individual target genes revealed that Aspn, Bgn, Dkk2, Ogn, Omd, and Ranbp3l may play roles in limiting VSV replication. The mechanisms by which these genes regulate VSV infection are not yet understood and will be the focus of future studies.

Our in vivo studies revealed that amiRNA-mediated increases in virus replication were tumor-specific. Since we did not identify the mRNA targets of the amiRNAs, we cannot speculate whether they are uniquely expressed in tumor cells. The tumor-specific increases in virus replication are likely a consequence of an already favorable cellular state for virus replication present in cancer cells. This environment enables robust virus replication and therefore high levels of amiRNA expression. In normal cells, virus replication is limited; thus, the levels of amiRNA expression are not high enough to impact virus replication. A similar scenario has been mathematically modeled to explain the tumor specificity of oncolytic VSVΔ51 expressing a soluble IFN-binding decoy receptor [4].

In summary, we have demonstrated the utility of our screening approach in generating more effective OVs. This approach may lead to the identification of amiRNAs that target cellular factors common to several cancer types, thus leading to the development of novel OVs as “off-the-shelf” therapeutics for certain cancers. However, in the context of personalized medicine, where one may envision passaging the library in a patient’s tumor sample and identifying amiRNAs that are specific to that patient’s cancer, there are still some unknowns. How rapidly an OV expressing an amiRNA could be identified, generated, validated, and approved for use in the same patient poses certain challenges in the clinic that will require ingenious solutions.

## 4. Materials and Methods

### 4.1. Cells

The CT26WT (murine colon carcinoma), BHK-21 (baby hamster kidney fibroblast), U2OS (human osteosarcoma), HCT116 (human colorectal carcinoma), and Vero (African green monkey kidney epithelial cells) cell lines were obtained from the American Type Culture Collection (ATCC, Manassas, VA, USA). Cells were cultured in Dulbecco’s modified eagle medium (DMEM; Hyclone, Logan, UT, USA) supplemented with 10% fetal bovine serum (FBS) (Hyclone) and cultured at 37 °C under 5% CO_2_.

### 4.2. Viruses

The SINV miR-30-based amiRNA library, composed of approximately 16,000 unique clones, has been described previously [19,56]. The cloning and rescue of recombinant SINV clones expressing GFP or amiRNAs have been described elsewhere [19,57]. Briefly, the SINV clones were created by cloning in amiRNAs into the TE12Q clone, a genetically modified strain containing a duplicate subgenomic mRNA promoter downstream of the structural genes [57]. amiRNAs were cloned by using existing MluI and NotI restriction sites. Plasmid DNA encoding the SINV–amiRNA viruses was subsequently transcribed by using the mMessage mMachine SP6 Kit (Ambion, Austin, TX, USA), and 6 μg of RNA was transfected into BHK-21 cells. Rescued virus was harvested 48 h post-transfection and propagated in BHK-21 cells.

The construction and rescue of recombinant strains of VSVΔ51 and MG1 have been described previously [7,15,44,58]. Briefly, the cloning of viruses expressing an amiRNA was achieved by the PCR amplification of the miR-30 cassette, by which XhoI and NheI sites were introduced into the PCR product. The PCR product was then digested and ligated into the pXN vector, and the rescue of recombinant viruses was performed as described previously [59,60]. All VSVΔ51 and MG1 stocks were propagated in Vero cells. For animal studies, VSVΔ51 stocks were further purified from cell culture supernatants by filtration through a 0.22 μm Steritop filter (Millipore, Burlington, MA, USA) and centrifugation at 30,000× *g* before resuspension in phosphate-buffered saline (PBS) (Hyclone). The VVdd-mCherry used in this study has been described previously [45]. Vaccinia stocks were propagated in U2OS cells, and cell-associated virus was collected by repeat (3) freeze–thaw cycles. Further purification of viral stocks was performed by centrifugation at 20,700× *g* through a 36% sucrose cushion (in 1 mM Tris) before resuspension in 1 mM Tris, pH 9.

### 4.3. SINV–amiRNA Library Screen

A total of 5 × 10^6^ CT26WT cells were infected at an MOI of 0.1 with the SINV–amiRNA library. This MOI ensured that all clones were sufficiently sampled. Supernatants were collected 48 h post-infection. The viral outputs from CT26WT cells were amplified on BHK-21 cells between passages in order to achieve an input MOI of 0.1 for the subsequent round of infection. A total of 4 passages in CT26WT cells was performed. In this study, amiRNA enrichment is defined as an increase in the frequency of a given amiRNA in the library after 4 rounds of passage relative to its frequency in the original amiRNA library.

### 4.4. Sequencing of SINV–amiRNA Library

The monitoring of viral populations has been described previously [19]. Briefly, random hexamers were used with Superscript II (Invitrogen, Carlsbad, CA, USA) to generate cDNA; then, specific primers with barcoded Illumina linkers were used to amplify the amiRNA hairpin region. Samples were analyzed on the Illumina HiSeq 2000 platform (San Diego, CA, USA). The amiRNA backbone was then trimmed, and distinct sequences were identified. Alignments were used to group sequences into “families” to control for sequencing error.

### 4.5. Cell Viability Assay

CT26WT tumor cells were infected with VSVΔ51, and cell viability was assessed by the alamarBlue (Life Technologies, Carlsbad, CA, USA) assay according to the manufacturer’s protocol.

### 4.6. Coomassie Blue Staining

Cells were fixed with a 3:1 mixture of methanol–acetic acid for 30min. The fixative was washed off with tap water, and cells were stained with a 0.1% solution of Coomassie Blue (Sigma-Aldrich, St-Louis, MO, USA) for 30 min. The stain was rinsed off with tap water, and plates were allowed to dry before imaging.

### 4.7. siRNA Transfection

Cells were transfected with 50 μM siRNA by using Lipofectamine RNAi MAX transfection reagent (Invitrogen), according to the manufacturer’s protocol. The next day, the cells were collected and re-plated in 12-well plates for infection. Cells were then infected with the indicated viruses 48 h post-transfection.

### 4.8. Quantification of Infection in Cultured Cells, Tumors, and Spleens

Tumors were excised from euthanized mice and homogenized in PBS. Homogenates or culture supernatants containing virus were serially diluted and plated onto Vero cells for 24 h for VSVΔ51 and MG1 plaque assays, Vero cells for 48 h for SINV plaque assays, and U2OS cells for 72 h for VV plaque assays.

### 4.9. Dual-Luciferase Reporter Assay

Lipofectamine2000 (Invitrogen) was used to transfect BHKs with 500 ng of psiCHECK-2 plasmid (Promega, Madison, WI, USA) containing the indicated target sites cloned in tandem in the 3′ UTR of Renilla luciferase. Two hours prior to transfection, BHK-21 cells were infected with VSVΔ51 expressing an amiRNA matching the target site in the 3′ UTR or with a control VSVΔ51 expressing an unmatched amiRNA (MOI of 5). Cell lysates were collected 24h post-infection, and the dual-luciferase reporter assay (Promega) was performed according to the manufacturer’s protocol. Renilla luciferase levels were normalized to Firefly luciferase and the unmatched amiRNA virus control.

### 4.10. Virus Competition Assay

amiRNA-expressing virus was mixed with control GFP virus in a ratio of approximately 1:100 and used to infect CT26WT cells at MOIs of 0.1 for SINV and 0.001 for VSVΔ51. Supernatants were collected 48 h post-infection, and virus titers were quantified by the plaque assay. For SINV, the outputs from CT26WT cells were amplified on BHK-21 cells between passages in order to achieve an input MOI of 0.1 for the subsequent round of infections. This was repeated for a total of 4 passages. The number of GFP-positive plaques was determined by selecting 100 well-isolated plaques at random and determining if they were GFP-positive or -negative by fluorescence microscopy.

### 4.11. Quantitative PCR

Cells were transfected with 50 μM siRNA by using Lipofectamine RNAi MAX transfection reagent (Invitrogen), according to the manufacturer’s protocol. Cells were infected with the indicated viruses 48 h post-transfection. Samples were collected 16 h post-infection for RNA extraction, which was performed by using the RNeasy kit (Qiagen, Hilden, Germany). cDNA was generated by using random primers and SuperScript Reverse Transcriptase III (Invitrogen). qPCR was performed with SYBR Select master mix (Applied Biosystems, Foster City, CA, USA) on a Rotor-Gene RG3000A instrument (Corbett Research, Sydney, Australia). Gene expression levels were determined by using the delta delta Ct method and normalized to GAPDH. Primer sequences can be found in Table S2. All primers were obtained from Integrated DNA Technologies (Coralville, IA, USA).

### 4.12. ELISA

Samples for the IFN-β ELISA were generated by infecting CT26WT cells that had been transfected with the indicated siRNAs for 48 h and subsequently infected with VSVΔ51-YFP at an MOI of 0.01. Culture supernatants were collected 24 h post-infection. The ELISA (R&D Systems, Minneapolis, MN, USA) was performed according to the manufacturer’s protocol.

### 4.13. Microarray

Cells were transfected with 50 μM siRNA by using Lipofectamine RNAiMAX transfection reagent (Invitrogen), according to the manufacturer’s protocol. Cells were infected with the indicated viruses 48 h post-transfection. Samples were collected 16 h post-infection for RNA extraction, which was performed by using the RNeasy kit (Qiagen). Samples were analyzed with the GeneChip Mouse Gene 2.0 ST Array (Affymetrix, Santa Clara, CA, USA), and data were processed with Transcriptome Analysis Console software (Affymetrix v3.0). The microarray data were deposited at Gene Expression Omnibus (GEO) and are available at https://www.ncbi.nlm.nih.gov/geo/query/acc.cgi?acc=GSE291763 (accessed on 6 May 2025) under accession No. GSE291763.

### 4.14. Mouse and Tumor Models

CT26WT tumors were established by the subcutaneous injection of 1 × 10^6^ cells into the hind flank of 8- to 10-week-old female balb/c mice (Charles River Laboratories, Wilmington, MA, USA). Palpable tumors (~ 4 mm in diameter) were treated after approximately 10–14 days. SINV and VSV were administered at doses of 1 × 10^8^ plaque-forming units (PFU) and 5 × 10^8^ PFU, respectively. Intravenous injections were administered in the tail vein. The formula used to calculate tumor volume is length × width^2^/2. All experiments were performed in accordance with the institutional guidelines of the review board for animal care (University of Ottawa).

## 5. Conclusions

Our study highlights the potential of the SINV–amiRNA library screening platform to identify artificial microRNAs that enhance OV replication in tumor cells. By using this approach, we identified and validated two amiRNAs that increase the replication of several clinical candidate OVs both in vitro and in vivo. Further studies are required to determine the cellular targets of the amiRNAs to better understand the mechanisms by which they regulate virus replication.

## Data Availability

The microarray data were deposited at Gene Expression Omnibus (GEO) and is available at https://www.ncbi.nlm.nih.gov/geo/query/acc.cgi?acc=GSE291763 (accessed on 6 May 2025) under accession No. GSE291763. The original contributions presented in this study are included in the article/Supplementary Material. Further inquiries can be directed to the corresponding author. The raw data supporting the conclusions of this article will be made available by the authors upon request.

## Supplementary Materials

The following supporting information can be downloaded at: https://www.mdpi.com/article/10.3390/ph18050708/s1, Table S1: Frequency of reads of the top ten most enriched amiRNAs in each replicate after four rounds of serial passage in CT26WT cells, Figure S1: SINV-expressing amiRNA viruses outcompete parental virus in HCT116 cells, Figure S2: siRNA-1 and -2 increase viral genome copies in infected cells, Figure S3: Dual-luciferase reporter assay, Figure S4: IFN-β production correlates with VSVΔ51 replication, Table S2: Primer sequences used in this study.
