# Supplementary material for: RNAi Screening in Tumor Cells Identifies Artificial microRNAs That Improve Oncolytic Virus Replication
Source: Pharmaceuticals (Basel). 2025 May 10;18(5):708. doi: 10.3390/ph18050708 (PMC12115315; doi:10.3390/ph18050708)
Supplement: Supplementary file 1 [file pharmaceuticals-18-00708-s001.zip › pharmaceuticals-3609887-supplementary.pdf]

Table S1

Table S1: Frequency of reads of the top ten most enriched amiRNAs in each replicate after four rounds of serial passage in CT26WT cells.

amiRNA ID	Sequence	Frequency of Reads (%)					
		P0	P4-R1	P4-R2	P4-R3	P4-R4	P4-R5
1	GTGGGAGAGGAGACATGTTGGC	0.0343	26.1698	34.9893	28.8833	58.5099	36.5393
2	ATGTTGTTGGAGAATAAGACAA	N.D.	39.8990	40.1095	4.2531	7.2182	4.4809
3	ATGCAGTATGACACTCTCTGCA	N.D.	2.4948	4.5950	8.2516	12.0567	9.3752
4	TTAACGTGACAGACATGGCGGC	N.D.	0.0058	0.2513	26.6349	0.0433	N.D.
5	AGGCACAGACAGGTGTCTTCCA	N.D.	N.D.	9.1190	1.7738	1.6772	4.5425
6	ATGAGCAAGAGAGATGGATGTC	N.D.	0.7116	1.2709	N.D.	1.4508	7.8865
7	ATTCCTTGGCTTCATTTGTGCT	N.D.	6.4415	0.3972	0.2905	3.9819	N.D.
8	GTTTCCACCTATGGCCACTGGT	N.D.	0.9696	0.9448	4.3227	1.2053	0.4803
9	TATTGAATGGAGGTCCTAAGGG	N.D.	N.D.	N.D.	N.D.	1.9710	5.7629
10	TTCGATGAACATGGATTCTGCA	N.D.	0.0238	N.D.	3.6999	0.0024	3.7873
11	TAAATGGGTCTGACACTTAGAA	N.D.	N.D.	N.D.	N.D.	0.0075	6.1047
12	TTGGTGTGGGTTCTGGTCAGCA	N.D.	0.2364	2.4662	N.D.	1.0219	2.2367
13	TTTCGGCTTCAGTTCACTCCGG	N.D.	0.9158	0.1483	0.1926	0.4143	3.2441
14	TGGCATTCTGAATGGACCTGCC	N.D.	4.5921	N.D.	N.D.	N.D.	N.D.
15	TGTGAACAGCATGGTTTGGCAC	N.D.	4.3619	N.D.	N.D.	N.D.	N.D.
16	TTTGACTCTGGCTATTAGTCCG	N.D.	N.D.	N.D.	N.D.	4.3512	N.D.
17	TTTCCTGAGGATATTATTGGGG	N.D.	N.D.	N.D.	N.D.	N.D.	4.2352
18	TACTGTAAAGATTTCAAGACCC	0.0250	1.5417	N.D.	1.8560	N.D.	N.D.
19	TTCTGCAGGAATGAGATCACCA	N.D.	N.D.	N.D.	3.2761	N.D.	N.D.
20	GTGGTGACGGAGAGGTAGGGCC	N.D.	N.D.	N.D.	2.6890	N.D.	N.D.
21	TTTGGCACTGAAGTCATGTCCA	N.D.	2.4590	N.D.	N.D.	N.D.	N.D.
22	GATTCATGTGGAACACCAACAC	N.D.	N.D.	N.D.	2.2136	N.D.	N.D.
23	TGTTGGATGCCTCTTCAGGCCC	N.D.	N.D.	N.D.	2.0613	N.D.	N.D.
24	ATGCAGTTGGATTGACTTGCCT	N.D.	N.D.	1.9530	N.D.	N.D.	N.D.
25	TTGCTCAATCGAGTTCAGGGCC	N.D.	1.7812	N.D.	N.D.	N.D.	N.D.
26	TTGGAAGTTGGATACCATGGAG	N.D.	N.D.	N.D.	N.D.	1.4934	N.D.
27	CAGCTCATACTGGAAATCTGGC	N.D.	N.D.	0.4907	0.0092	N.D.	N.D.
28	TTGCAGCAGCATGGGAGTGCCC	N.D.	N.D.	0.4452	N.D.	N.D.	N.D.

P: passage ; R: replicate ; N.D.: not detected

Figure S1

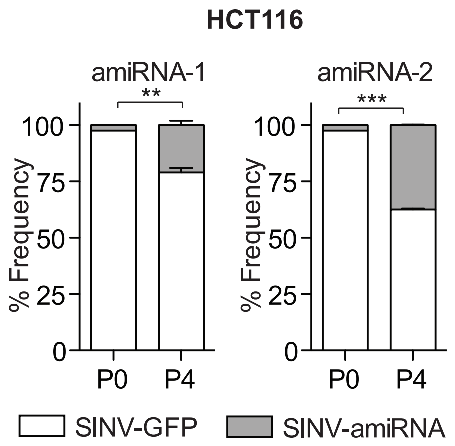

Supplemental Figure S1: SINV-expressing amiRNA viruses outcompete parental virus in HCT116 cells. Enrichment of amiRNA-expressing of SINV from competition experiments against parental virus SINV-GFP. The percentage of each virus is shown before competition (P0) and after 4 rounds of competition (P4) (n=2). Data are presented as means ± SEM. Student's unpaired, one-tailed t-test was performed; **: $p < 0.01$, ***: $p < 0.001$.

Figure S2

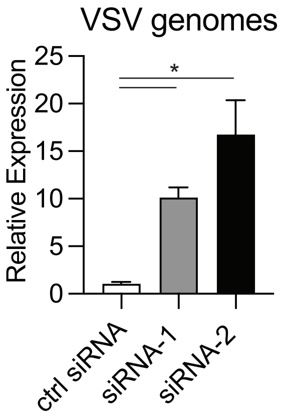

Supplemental Figure S2: siRNA-1 and -2 increase viral genome copies in infected cells. RT-qPCR analysis of VSV genomes following VSV infection of siRNA-treated CT26WT cells (n=3). Data are presented as means ± SEM. Student's unpaired, two-tailed t-test with Welch's correction was performed; *: $p < 0.05$.

Figure S3

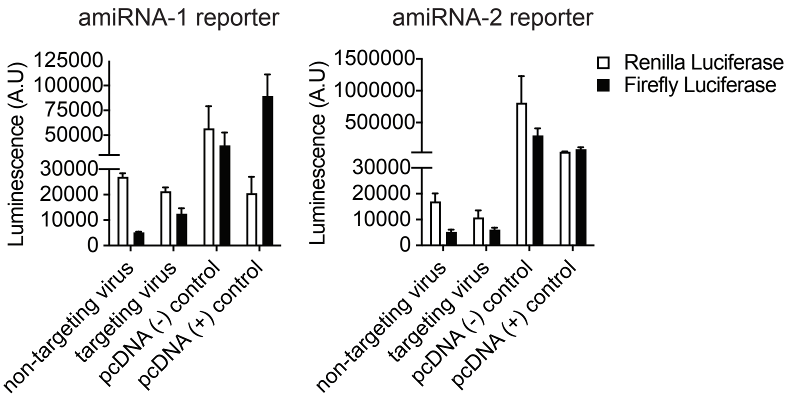

Supplemental Figure S3: Dual-luciferase reporter assay. BHK cells were transfected with the indicated reporter plasmids possessing target sites for the enriched amiRNAs in the 3' UTR of the Renilla luciferase reporter and subsequently infected with the indicated VSV viruses. Renilla and Firefly luciferase activity was measured 24h post-infection. Raw, non-normalized luminescence values are shown (n=3).

Figure S4

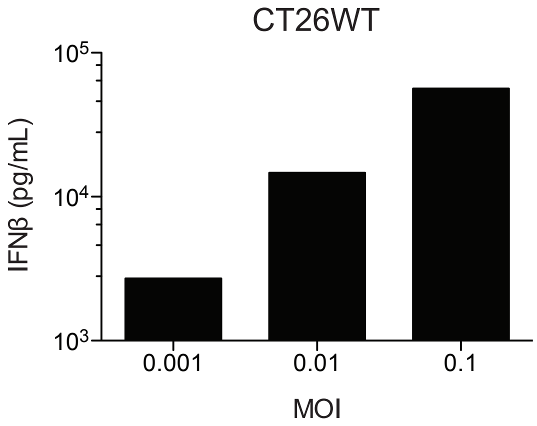

Supplemental Figure S4: IFN-β production correlates with VSVΔ51 replication. CT26WT cells were infected with VSVΔ51 at the indicated MOIs and IFN-β concentrations in the culture supernatants 24h post-infection were measured by ELISA (n=1).

Table S2

Table S2: List of primers used for qPCR analysis.

Gene Name	Forward Primer (5'-3')	Reverse Primer (5'-3')
**APLN**	GTGGATCCTGACATGGTTCTATAC	GTCCTTTGGGCTCTGACTTT
**ASPN**	GATACAAAAGGACACGTTCAAGG	GAGGTTAGTTTTGCTTCAGCG
**DKK2**	GAAGAATGAGGGATGTGGTAAGA	CATAACGGAAGCACTGGTAGTA
**GAPDH**	ACCACAGTCCATGCCATCAC	TCCACCACCCTGTTGCTGTA
**IFI44**	TCCATCCCTTTAGAGTGCTA	GCAAGCAGAACTAAGCTCAT
**IFIT3**	TCTCAGAAGCTCAGGCTTAC	ACATTTTTGCTCGTTCATTT
**IFN-β**	GATGACGGAGAAGATGCAGAAG	ACCCAGTGCTGGAGAAATTG
**MX2**	ATCAATGCTTTTAACCAGGA	TTTTCGATCTCCTTACTCCA
**OASL2**	TAAGCTGCTTTTCCAGTTTC	AGGACAATGATGTTGTAGGC
**OGN**	AGCCCAAGAGCACCATTT	CACCTTCCTCTGAAGCTTAGTT
**OMD**	ATCCAGTACACCATCACCATTT	GTGTATACGAGGGAAGCAGAAG
**VSV**	GATAGTACCGGAGGATTGACGACTA	TCAAACCATCCGAGCCATTC
